# The Impact of Systemic Insecticides: Cyantraniliprole and Flupyradifurone on the Mortality of *Athalia rosae* (Hymenoptera: Tenthredinidae) Based on the Biophoton-Emission of Oilseed Rape

**DOI:** 10.3390/insects16010035

**Published:** 2025-01-01

**Authors:** Bálint Gerbovits, Ildikó Jócsák, Sándor Keszthelyi

**Affiliations:** Department of Agronomy, Hungarian University of Agriculture and Life Sciences, Kaposvár Campus, S. Guba Str. 40, H-7400 Kaposvár, Hungary; jocsak.ildiko@uni-mate.hu (I.J.); ostrinia@gmail.com (S.K.)

**Keywords:** insecticides, systematic, *Athalia*, larvae, biophoton emission, non-invasive method, photosynthetic activity, turnip sawfly

## Abstract

The selection of an appropriate and targeted crop protection technology for winter oilseed rape is crucial for the economic production of this crop. Insecticides belonging to the group of diamides and butenolides are available as seed treatments for winter oilseed rape and serve as effective tools for chemical crop protection. The objective of this study was to determine the multi-directional applicability of the active ingredients cyantraniliprole and flupyradifurone. Furthermore, the phenotypic detection of stress was conducted for these systemic insecticides. The results of our study show that these active ingredients acropetal and basipetal translocation are both effective against *Athalia rosae* (L. 1758) (Hymenoptera: Tenthredinidae). The findings from our laboratory studies indicated that the insecticidal active ingredients are effective against *Athalia rosae* (Hymenoptera: Tenthredinidae) via both acropetal and basipetal translocation. However, differences in the time of exertion of lethal effect were recorded. In both experimental groups, mortality was observed to reach 100% at the 96-h mark, regardless of the specific experimental conditions or the insecticide employed. An examination of the bio-photon emission of the experimental plants revealed a statistically verifiable correlation between the applied active ingredients and the photon emission intensity per unit plant surface area. The findings of our study indicate that due to their insecticidal activity, systemic insecticides also affect plant life processes by increasing photosynthetic activity per unit leaf area.

## Simple Summary

The right crop protection technology is essential for winter brassica production. Brassica is one of Europe’s most intensive arable crops, with more than ten crop protection treatments in a season. Insecticides from the diamide and butenolide groups are available as seed treatments for winter oilseed rape. They are effective tools for chemical crop protection. We wanted to see how well the insecticides cyantraniliprole and flupyradifurone worked in different situations. We also looked at how these systemic insecticides affect the insects. Our lab studies showed that the insecticides were effective against *Athalia rosae* (L. 1758) (Hymenoptera: Tenthredinidae) when moved up and down the plant. However, there were differences in how long it took for the insects to die. For both insecticides, all the insects died after 96 h, no matter the setting or insecticide used. Our study showed that insecticides affect plant life processes by increasing photosynthetic activity.

## 1. Introduction

Winter oilseed rape (*Brassica napus* spp. oleifera L.) is regarded as the most significant oilseed crop globally, particularly in Europe. It is the third most widely cultivated oil crop in the world, with 26.5 million hectares under production, after oil palm and soybean. In Europe, it is the most extensively cultivated oilseed crop, with 11.6 million hectares under cultivation [1]. Winter oilseed rape is attacked by a wide range of arthropods, for which plants of the Brassicaceae family are their main food source. [2]. These species are considered oligophagous, and therefore insecticidal control is a mandatory element in the cultivation of this crop. The successful and economical cultivation of these oilseed crops depends on the judicious and targeted choice of effective crop protection technologies. Failure to adopt successful control procedures will have a significant impact on the success of the sector and, in turn, on the success of oilseed rape production [3].

The turnip sawfly, *Athalia rosae* (L. 1758) (Hymenoptera: Tenthredinidae), is a well-documented and economically significant pest within the oilseed rape pest community in Europe. The primary damage is caused by the larva, which possesses a powerful chewing mouthpart. The species completes two to three generations per year, overwintering as larvae within damaged plant tissue and in various hiding places, including the protective sheaths it creates. The larvae pupate in the spring, and the adults start to draw from the second half of April [4]. The female imago lays her eggs in the phloem part of the leaf, from where the larvae hatch, and takes 3 weeks to reach the most advanced larval stage (L_6_). It is anticipated that the emergence and subsequent damage will occur by the end of October. Annually, the presence of *A. rosae* larvae can result in considerable issues for winter oilseed rape cultivation regions. The larvae emerge from their eggs and initially display a colouration similar to that of the leaves. They subsequently chew small, round holes in the leaves, thereby physically destroying the plant tissue. In the absence of adequate protection, the older larval stages have the potential to skeletonise or even completely chew the oilseed rape, ultimately leading to the death of the plant [2,3,4].

The European Union currently has a wide range of pesticides available for the successful control of pests of winter oilseed rape. However, the insecticides most commonly used in the control of this crop are primarily non-systematic, and only a limited number of absorbable active ingredients have been granted authorisation for use in seed dibbing and crop management. The active ingredients insecticide ingredients are classified according to the Insecticide Resistance Action Committee Mode of Action (IRAC MoA). The most promising active ingredients include cyantraniliprole, a compound belonging to the group of diamides (IRAC MoA:28), and flupyradifurone, an insecticidal active substance belonging to the group of butenolides (IRAC MoA:4D) [5]. These products are available for use in oilseed rape cultivation in both crop treatment and seed treatment. These absorbable insecticidal active ingredients are distinguished by a multidirectional translocation pathway within the plant transport tissue system [6,7]. The translocation occurs in both an acropetal direction via xylem elements and a basipetal direction via phloem elements, thus ensuring the efficient reaching of their sites of action for both seed dehiscence and stocking [7,8,9]. The cyantraniliprole zoocidal agent that affect nerve and muscle function. The regulated release of calcium from the intracellular space into the cytoplasm of the cell is essential for muscle function [10]. The anthranilic acid derivative diamides activate insect ryanodine receptors (RyRs), which play a significant role in striated muscle function [11,12,13]. Upon binding to RyRs, diamide molecules result in an unregulated release of calcium, effectively halting insect locomotion and feeding shortly after exposure [10]. It has been demonstrated to be highly efficacious against a range of pests, including both chewing pests (such as beetles and beetle and moth larvae) and sniffer pests (such as bedbugs, aphids, and thrips) [7,12]. The active substance flupyradifurone is a compound belonging to the group of zoocidal agents that act on neurons, specifically the butenolides. As outlined by Nauen et al. [6], flupyradifurone initially stimulates the nerve endings of insects, resulting in the overactivity of striated muscles and subsequent paralysis of the muscles within a relatively short time. The mechanism of action of this active substance is analogous to that of neonicotinoids and sulfoxamides, whereby they bind to nicotinic acetylcholine receptors (nAChR) in insect nerve cells, thereby inducing a continuous state of excitation. Chen et al. [14] demonstrated remarkable efficacy against sniffer pests (Hemiptera: Aphididae, Aleyrodidae, Psyllidae) due to the rapid neurostimulatory effect of the compound [6,15,16]. The active ingredients cyantraniliprole and flupyradifurone are systemic compounds that are absorbed into the plant and are characterised by multidirectional (acropetal, basipetal) translocation within the plant transport tissue system [6,7]. This makes them both excellent for use as seed treatment and as stock treatments. Several international studies [3,17,18] have reported on the severity of oilseed rape damage to cruciferous crops, which ultimately determines the economic viability of oilseed rape production.

Hideg et al. [19] have demonstrated that metabolic products of plant cell organelles can induce chemical reactions that result in root formation. These highly active compounds are capable of emitting light. As stated by Rastogi et al. [20], the phenomenon whereby light is emitted as a consequence of chemical reactions occurring in living organisms is referred to as bioluminescence. This process is the result of oxidation processes, and due to its ultra-low intensity, bioluminescence is invisible to the naked eye [21]. However, it can be detected, recorded, and measured by a camera with sufficient sensitivity [22]. Lukács et al. [23] posit that plants are capable of releasing unused energy in the electron transport chain of photosystem II in the form of photon emission in addition to heat emission. In the absence of light, the photosynthetic process is terminated, and some of the electrons that have not been utilised in the electron transport chain are returned to the reaction center. Here, chlorophyll molecules are excited and emit photons in the red light wavelength. Following the findings of Sánchez-Moreiras et al. [24], this phenomenon is referred to as delayed fluorescence (DF). As stated by Jócsák et al. [22], this phenomenon is indicative of the status of the photosynthetic apparatus. It has been demonstrated by several researchers that DF measurements can be used to determine plant stress phenomena in vivo [23,24]. The emission of photons from oxidative metabolic processes in plant cells is a phenomenon known as ultraweak bioluminescence (UWLE). Photon emission has been observed in some enzymatic processes, including those occurring in mitochondria and peroxisomes. It has also been demonstrated to occur during the formation of reactive oxygen species (ROS), which can result in non-specific processes in lysates and membrane proteins. Consequently, the process of lipid oxidation results in an increase in UWLE [22,25,26].

The principal objective of our study was to ascertain the time-dependent mortality-inducing effects of the insecticides cyantraniliprole and flupyradifurone on *A*. *rosae*, employing objective methodologies. Furthermore, biophoton emission analyses were conducted to detect plant stress phenotypically, thereby enabling the determination of the changes in plant metabolism induced by the active ingredients and insect damage. Consequently, employing this contemporary non-invasive imaging technology, our objective was to map the applicability of the aforementioned active ingredients against this chewing mouthpart pest in a unique way.

## 2. Materials and Methods

### 2.1. Experimental Parameters and Settings

**Figure 1:**
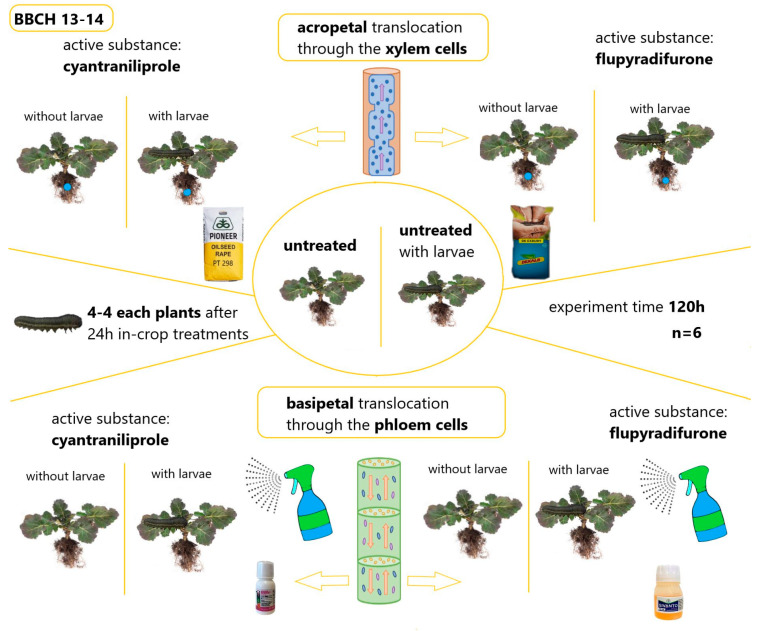
Types of treatments used in an experiment against *Athalia rosae*.

The study employed three distinct seeds, which were applied to both the in-crop-treated and seed-treated plants. The experimental variety was DK Exbury^®^, which is uncoated. The hybrids DK Exbury^®^ and Pioneer PT298^®^ winter oilseed rape were employed in the seed stripping samples. The active ingredients employed were cyantraniliprole (Lumiposa^®^ FS, 625 g/L active ingredients, Corteva Agriscience^TM^, Indianapolis, IN, USA) and flupyradifurone (Buteo Start^®^ FS, 480 g/L active ingredients, Bayer Crop Science, Monheim am Rhein, Germany). The plants were germinated in vitro in pots measuring 10 × 8 cm in September 2023. A single seed was planted in each pot at a uniform depth of 2 cm to ensure homogeneous germination and uniform plant development. The soil was Florimo^®^ general potting soil, comprising a minimum of 70% organic matter and a pH of 6.4. No supplementary nutrients were applied. The efficacy of the active ingredients cyantraniliprole and flupyradifurone were evaluated for seed treatment and crop spraying applications, with the objective of mapping acro- and basipetal translocation. To assess the efficacy of these insecticides, a sample of mixed-sex *A*. *rosae* larvae was collected from pesticide-free environments. The collection was conducted in the final days of August 2023 in the vicinity of Karád, Hungary (Somogy County; GPS coordinates: 46°69′07.60″ N 17°84′13.60″ E). Specimens were collected that were observed to exhibit high levels of mobility and pronounced feeding activity on plants that flowered in flood conditions and on second-seeded cruciferous plants. The larvae were maintained under isolator conditions until the commencement of the experiment. The experimental plant population was cultivated until it reached the three-to-four-lobed leaf stage (BBCH 13–14). The study employed five distinct treatments, with six replicates each (Figure 1):

1. untreated;
2. cyantraniliprole seed treatment;
3. cyantraniliprole in-crop treatment;
4. flupyradifurone seed treatment;
5. flupyradifurone in-crop treatment.

The doses of the active substance employed in the seed treatment were 16.00 l a.i. t^−1^ for cyantraniliprole and 10.42 l a.i. t^−1^ for flupyradifurone, as delineated in the authorisation dossier. The active ingredients were administered with under the specifications outlined in the authorisation dossier for these preparations, with doses of 0.625l a.i. ha^−1^ employed in-crop treatment. The quantities of insecticides applied and used were determined by the recommendations set forth by Bayer Crop Science and Corteva Agriscience™. *A. rosae* larvae were released 24 h after the treatments were applied, utilising an isolator on the experimental crop. For each experimental setup, 6 plants were used, with 4-4 larva per plant. Stock sprays were conducted at the outset of the experiment, after which the mortality induced by the active ingredients under examination and the detection of plant stress phenotypes were monitored and evaluated over five days. During the experiment, the plant population was placed in an incubator outside of the designated measurement and recording period. The conditions established within the incubator (18 °C temperature, 15:9 L:D photoperiod, and 60% relative humidity) replicated the seasonal environmental conditions that would be typical for Hungary during that period.

### 2.2. Non-Invasive Imaging Methodology

The NightShade LB 985 in vivo plant imaging system (Institute Berthold Technologies Bioanalytical Instruments, Calmbacher Strasse 22, D-75323 Bad Wildbad, Germany) was employed for the measurement and detection of biophoton emission. This instrument is equipped with a slow-scanning, sensitive, thermoelectric Peltier-cooled NightOwlcam CCD (charge-coupled device) for the purpose of bioluminescence measurements. Furthermore, the camera of this device is utilised in the field of astronomy, with the capacity to detect the light of a single candle from a distance of up to 10 km. The instrument is operated via IndiGo™ 2.0.5.0 software (Institute Berthold Technologies Bioanalytical Instruments, Calmbacher Strasse 22, D-75323 Bad Wildbad, Germany). As the set-up parameters were maintained throughout the recordings, the alterations in relative pixel intensities reflected the intensity of the photon emissions generated by the treatment or insect damage. The resulting values were determined using the IndiGO™ software and expressed as CPS (counts per second) in order to accurately determine the photosynthetic activity per unit plant area. The integration time was set to 20 s, utilising a 4 × 4 pixel combination. A simultaneous background correction and cosmic ray attenuation were performed during image acquisition to eliminate the influence of high-intensity gamma rays, which were deemed to be irrelevant. For each measurement, bioluminescence was immediately assessed following the plants’ placement in the dark chamber. The delayed fluorescence (DF) signal was then measured and recorded for period of 15 min. Subsequently, during the five-day experiment, one-hour measurements were conducted to assess the oxidative metabolism of the experimental plants, with the results recorded from min 45 to min 60. The evaluation from the 45th min served to ensure that the signals from photosynthesis did not distort the ultraweak-bioluminescence values.

Furthermore, the study encompassed the extent of damage sustained by larvae over 120 h. This was conducted utilising GIMP 2.10.32 pixel analysis software (GIMP Development Team, University of California, Berkeley, CA, USA). During the evaluation, the plants that had not been treated with insecticides, and thus larvae that were intensively feeding on oilseed rape, were analysed. This was done in order to determine the extent of damage caused by *A. rosae* to the experimental plant population every 24 h.

### 2.3. Evaluation and Statistical Analysis

A Shapiro–Wilk [27] analysis was employed to examine the mortality data of *A*. *rosae*, with a sample size exceeding 50. In order to assess the normality of the distribution of the data, the Ghasemi and Zahediasl [28] methods were employed. This was done to ensure that the *p*-value was less than 0.05. The data were analysed using a one-way ANOVA test in SPSS 29.0 software, with consideration given to the effects of insect mortality, time since treatments, and the different application methods of the flupyradifurone and cyantraniliprole active ingredients. The resulting values were then subjected to a Tukey’s (HSD) test for separation. The Abbott [29] correction was employed to ascertain the mortality values.

## 3. Results

### 3.1. Effect of Active Ingredients on Insect Mortality

**Figure 2:**
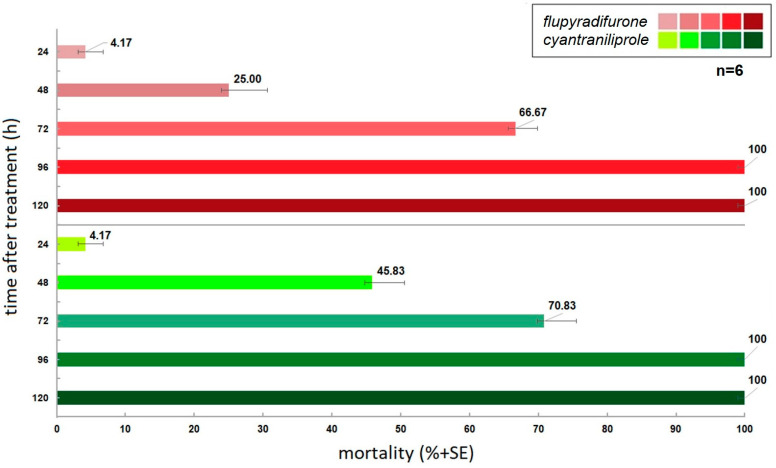
Abbott corrected mortalities triggered by examined insecticide active ingredients as an in-crop application mode.

The results of our laboratory study demonstrate that the tested and analysed cyantraniliprole and flupyradifurone insecticides are effective against the larvae of the *A. rosae*. However, a fundamental distinction was observed in their capacity to induce mortality (Figure 2). About the mortality-inducing effects of the various active ingredients, the stock treatment of cyantraniliprole demonstrated the most rapid and pronounced impact on insect viability. A total mortality of 100% was observed after 96 h from the commencement of the experiment. The highest mortality rate was observed three days following treatment with the active substance, with mortality values exceeding 45% on the second day and 70% on the third day in the experimental insect population. The insecticidal effect of flupyradifurone, a member of the butenolide group of active ingredients applied during the herd treatment, was observed to be slightly less pronounced. While absolute mortality was also documented for this treatment at the 96 h mark, the insecticidal activity on the second day of the experiment was approximately half of the values recorded for cyantraniliprole (25%), while on the third day, the basipetally transported insecticide approached the mortality values generated by cyantraniliprole but yielded 4% lower values (67%). Furthermore, absolute mortality was also recorded for flupyradifurone 96 h after exposure.

**Figure 3:**
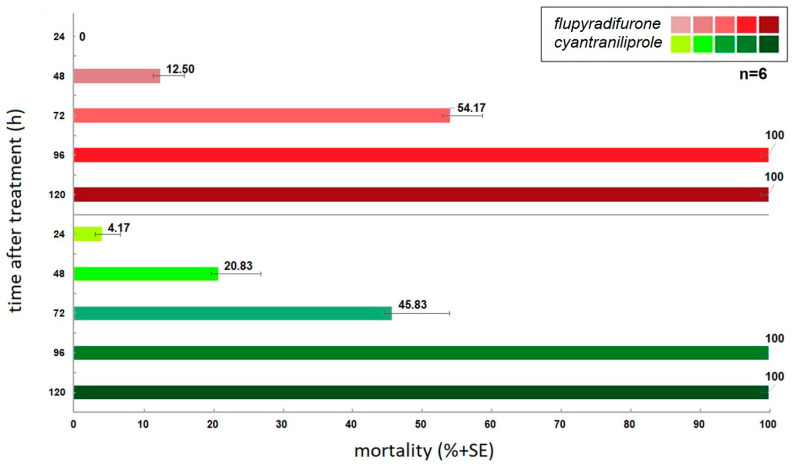
Abbott corrected mortalities triggered by examined insecticide active ingredients as a seed treatment application mode.

The results of this experiment demonstrated a discernible distinction in the efficacy of the various application methods (Figure 3). The insecticide active ingredients applied by seed treatment demonstrated mortality values that were comparable to one another but exhibited disparate trends. Of the two active ingredients, the acropetal translocation cyantraniliprole exhibited shorter mortality rates, with 4% and 21% mortality recorded on the first two experimental days. In contrast, the acropetal translocation flupyradifurone was the sole experimental setting in which mortality was not observed 24 h after the release of the insects. However, it did produce mortality values exceeding 54% on the third day of the experiment, which exceeded the insecticidal effect of cyantraniliprole, which produced higher values initially. The acropetal translocation of both active ingredients generated a clear 100% mortality on the fourth day of the experiment.

The results of our observations corroborate the hypothesis that insect individuals exhibiting sensitivity to the insecticide treatment cease to move with great intensity and demonstrate a reduction in feeding activity. However, no alterations in behaviour or feeding patterns were discerned in individuals that had not undergone the insecticide treatment, irrespective of stocking or seed treatment conditions. The presence of overwintering larvae was confirmed five months after the conclusion of the experiment (1 February) in control individuals of the experimental insect population. Table 1 shows the mortality results of statistical analysis.

### 3.2. The Extent of Chewing the Oilseed Rape Leaves

**Figure 4:**
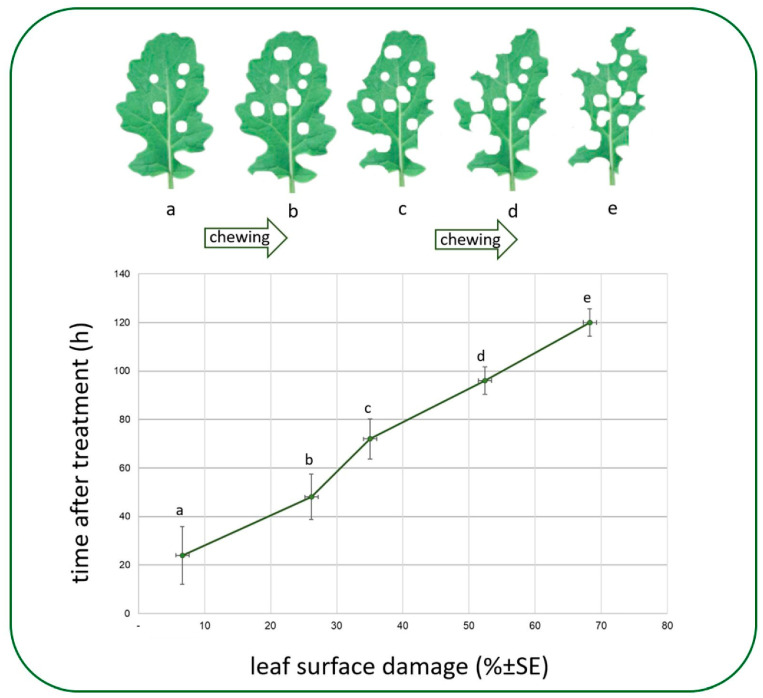
The extent of chewing the oilseed rape leaves in 120 h by *Athalia rosae* larvae.

Our studies have essentially validated the assertion that *A. rosae* represents one of the most detrimental pests of winter oilseed rape, as it can inflict a considerable diminution in leaf area during the initial juvenile phase of the plant. The results demonstrate that approximately 7% of the photosynthetic leaf area was destroyed within 24 h following the release of the larvae. The insects fed intensively on the experimental plant population throughout the study, using their powerful chewing mouthparts. After 96 h, more than half (52.4%) of the leaf area of the test plant population had been destroyed. After five days of intensive feeding, on average, nearly 70% of the photosynthesising leaf area had been destroyed by the experimental insect population (Figure 4).

### 3.3. Results of Delayed Fluorescence Response to the Different Application

**Figure 5:**
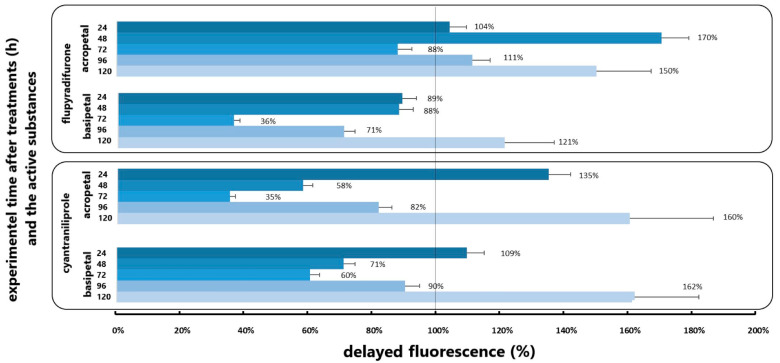
Delayed fluorescence values generated by oilseed rape plants were tested without damaging the larvae. The values were expressed as % of the untreated plant values.

The biophoton emission analysis corroborated the alterations in plant metabolic responses to the insecticidal active ingredients cyantraniliprole and flupyradifurone that were subjected to testing. The alterations in the analysed pesticides in plant metabolic responses are illustrated in Figure 5. The values plotted here are in relation to the control group, whereby an excess of 100% of the control group value indicates stress-induced changes in the plant.

In the absence of insect individuals, significant differences were observed in the delayed fluorescence phenotype of rapeseed plants treated with the acropetally transported flupyradifurone active substance at 24, 48, 96, and 120 h post-experiment. These values were significantly higher than those recorded for the untreated plants. About this active ingredient, the greatest increase in DF values was observed at 48 h post-experimental introduction, with the photon emission values of treated plants exceeding those of the control by 70%. When translocated basipetally, the same active substance demonstrated an increase in delayed fluorescence intensity solely at the 120th hour of the experiment, resulting in lower values compared to the control plants on the other days (Figure 5). In contrast, the applied cyantraniliprole active substance yielded a more moderate result, as it was transported acropetally and basipetally, demonstrating significant differences compared to control plants on the first and last days of treatment. In this instance, the acropetal method yielded a 35% increase in growth after 24 h, whereas the basipetal method resulted in a 9% increase. On the fifth day of the experiment, the cyantraniliprole insecticide exhibited nearly equal increases in photon emission intensity when transported acropetally and basipetally, reaching approximately 60% in both cases. The statistical analysis revealed no statistically significant differences (Table 2) in the increased delayed fluorescence values generated by the active ingredients under examination (*p* > 0.05).

**Figure 6:**
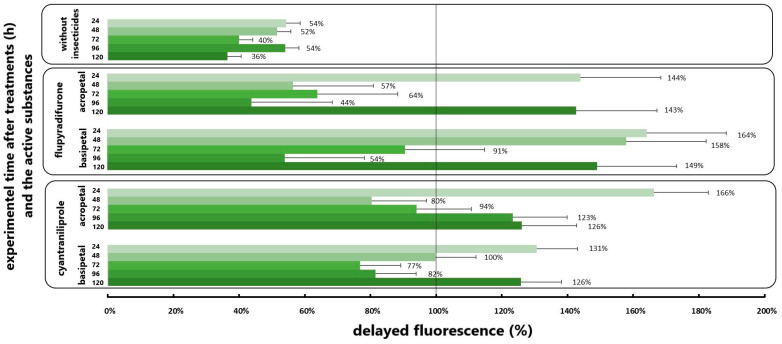
Delayed fluorescence values generated by oilseed rape plants were tested with damaging the larvae. The values were expressed as % of the untreated plant values.

In the case of examining the combined biophoton emission of plants treated with the analysed active ingredients and those damaged by the *A*. *rosae* larvae, an increase in photosynthetic activity of the plants was observed (Figure 6). The application of insecticides to rapeseed plants that had not previously been treated with insecticides but were exposed to the chewing of the larvae resulted in a significant reduction in the values of delayed fluorescence in comparison with those observed in intact plants. In this experimental setting, the values generated by the control plants were approximately 50% of those generated by the control plants at all time points tested and analysed. In the case of oilseed rape plants exposed to damage caused by *A. rosae* and treated with the active ingredient flupyradifurone, a significant increase in the change in delayed fluorescence was observed at 24, 48, and 120 h of the experiment. In this experimental setting, the combined biophoton emission of the plants was found to exceed that of the untreated plants by an average of 57% at the aforementioned time points. The basipetal translocation of the same active substance demonstrated a comparable, statistically significant increase at multiple time points. In contrast, an examination of the combined effect of the cyantraniliprole insecticide on larval damage revealed an increase in the change in delayed fluorescence in both experimental settings. The increases demonstrated a statistically significant distinction between the initial and final days of the experiment for cyantraniliprole administered as seed treatment and as an in-crop treatment (Figure 6).

The analysis revealed significant differences between oilseed rape plants treated with the insecticide active ingredient and those not treated with the active ingredient. Concurrently, the statistical analysis indicated that there was no statistically significant difference between the active ingredients under consideration, with a *p*-value of 0.05 or greater (Table 2).

### 3.4. Results of Ultraweak Bioluminescence

**Figure 7:**
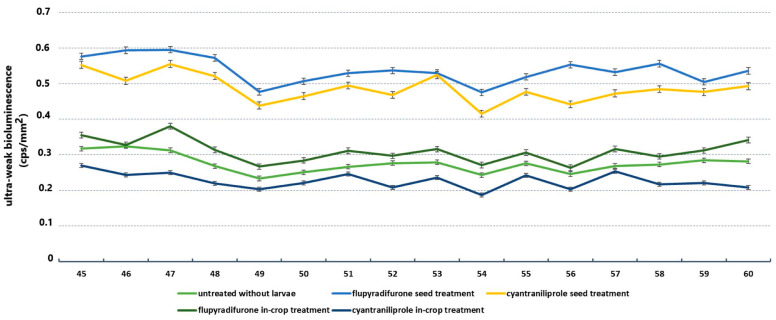
Ultraweak bioluminescence values generated by oilseed rape plants without *Athalia rosae* larvae.

For the mapping of oxidative processes, ultraweak bioluminescence (UWLE) values were taken from 45 to 60 min after insertion into the instrument (Figure 7). The measured UWLE values for plants treated with insecticides without damaging pseudomonas were higher than the mean for untreated plants in three experimental settings (0.28 ± 0.013). The highest increases were observed for cyantraniliprole and flupyradifurone applied as seed treatments. The mean of this increment for flupyradifurone acropetal UWLE was 0.54 ± 0.018, while the mean for acropetally delivered cyantraniliprole was 0.49 ± 0.02. This represents an increase of 50% for flupyradifurone and 45% for cyantraniliprole. In addition, an increase in measured oxidative processes was observed for the active ingredient flupyradifurone applied as an in-crop treatment, with a mean of 0.31 ± 0.016. Uniquely, the active ingredient cyantraniliprole applied basipetally showed a lower UWLE value compared to untreated plants, with an average value of 0.23 ± 0.011. The statistical analysis of the investigated parameters reveals significant differences in the ultraweak bioluminescence values generated by the active ingredients under consideration. The results of the statistical analysis are presented in Table 3 for the reader’s convenience.

**Figure 8:**
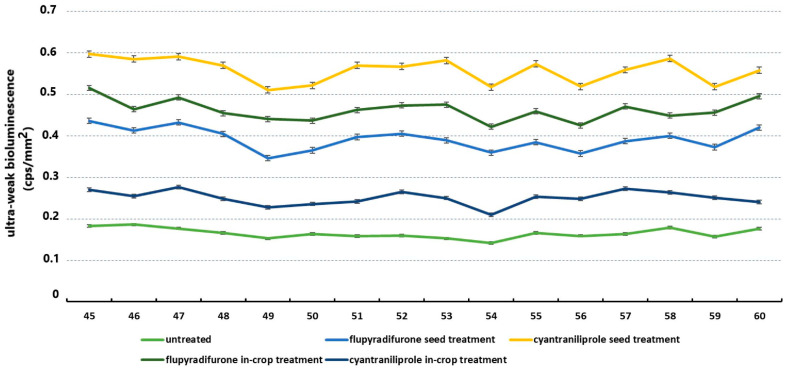
Ultraweak bioluminescence values generated by oilseed rape plants damaged by *Athalia rosae* larvae.

In all cases, the UWLE values of plants exposed to larval damage and treated with the tested insecticide active ingredients exceeded those of plants not treated with the active substance but damaged, generating an average value of 0.17 ± 0.006. In the case of oilseed rape plants damaged by the *A. rosae* of oilseed rape, plants seed-treated with the active substance cyantraniliprole exhibited the highest ultraweak bioluminescence values of 0.56 ± 0.015. A trend effect was observed for the UWLE values of the experimental plants, as the results generated by the damaged but untreated plants exceeded those generated by the untreated plants by almost 10 values in each experimental setting, in step one. Accordingly, basipetal delivery of cyantraniliprole yielded 0.25 ± 0.009, acropetal application of flupyradifurone produced 0.39 ± 0.013, and flupyradifurone applied as an in-crop treatment resulted in 0.46 ± 0.012. Conversely, acropetal delivery of cyantraniliprole demonstrated 0.56 ± 0.015 in the assessment of oxidative metabolism (Figure 8).

The statistical analyses of all experimental setups revealed significant differences in the parameters under investigation. Table 3 presents the statistical results pertaining to the UWLE values generated by *A. rosae* larvae.

## 4. Discussion

The laboratory analyses confirmed the multidirectional transport of the analysed cyantraniliprole [7,30] and flupyradifurone [6,11] active ingredients within the plant transport tissue system, as previously described in international studies. The experiments and subsequent analyses yielded absolute efficacy and 100% mortality for all samples included. The most rapid and pronounced insecticidal effect was observed in the case of the active substance cyantraniliprole, when administered as a herd treatment. The efficacy of this diamide molecule against chewing mouthpart pests has been previously documented by Pes et al. [7] in their investigation of the bean seed fly (*Delia platura* Meigen, 1826) and the European corn borer (*Ostrinia nubilalis* Hbn., 1796). In a recent study, Keszthelyi et al. [31] demonstrated the long-lasting residual efficacy of the active substance cyantraniliprole against chestnut leaf miner larvae. In contrast, flupyradifurone demonstrated a more moderate outcome in the mortality of *A. rosae* larvae during the initial three days of the experiment. This may be attributed, among other factors, to the fact that this active ingredient exhibits the highest efficacy against the sniffer (Hemiptera, Aphididae) pest, as previously confirmed by Chen et al. [14] against *Diaphorina citri* Kuwayama, 1908. Furthermore, outstanding activity against sniffer pests was also reported by Roditakis et al. [32], whereby the toxic effect of the active ingredient flupyradifurone on the rapid nervous system was demonstrated to be an effective method against *Bemisia tabaci* Gennadius, 1889. Additionally, it prevented the transmission of tomato yellow leaf curl virus vectored against sniffer pests. In light of the aforementioned findings, it can be posited that the active ingredient cyantraniliprole displays augmented efficacy against chewing mouthpart pests when administered basipetally, in comparison to the insecticidal active ingredient flupyradifurone. In contrast, Barry et al. [9] investigated the multidirectional translocation of the active substance cyantraniliprole and demonstrated that this active substance did not exhibit significant activity against *B*. *tabaci*, *Plutella xylostella* L., 1758, and *Myzus persicae* Sulzer, 1776, via the phloem elements.

The insecticidal active ingredients were tested during seed treatment and yielded more moderate results in mortality values measured as a function of time across xylem elements. During the acropetal delivery of the two active ingredients, cyantraniliprole exhibited a significantly higher mortality value over a shorter time period. The acropetal applicability of this active substance against chewing mouthparts was confirmed by Keszthelyi et al. [31] in their study on *Cameraria ochridella* Deschka and Dimic, 1986. Molnár et al. [33] reported that the active ingredient cyantraniliprole exhibited outstanding acropetal properties against *Delia radicum* L., 1758, a pest that causes significant damage to oilseed rape. Barry et al. [9] provided evidence of the acropetal transport of the active ingredient cyantraniliprole via the translocation process through xylem elements. In their study, the target pest, *Helicoverpa zea* Boddie, 1850, exhibited sensitivity to the insecticide, resulting in significant mortality. In the initial two-day period of the experiment, the efficacy of flupyradifurone when applied acropetally was observed to be lower than expected. In consideration of the basipetal values, our findings indicate that the neurotoxic impact of the active substance flupyradifurone on the chewing mouthparts is temporally delayed, in contrast to the effect of the active substance cyantraniliprole, a member of the diamide group, on RyRs receptors, which regulate nerve and muscle function. In a laboratory study, Stamm et al. [34] demonstrated that this active substance exhibits a rapid initial acropetal translocation. The exceptional acropetal property of flupyradifurone was documented by Gerbovits et al. [16] in *Eurydema ventralis* Kolenati, 1846, resulting in mortality in nearly 71% of insects within 120 h. The efficacy and multidirectional translocation of flupyradifurone as an active ingredient against pests have also been corroborated [14,32].

The environmental friendliness of these third-generation insecticidal active ingredients has been reported by Lahm et al. [10], Campbell et al. [35], Fang et al. [8], and Hasselbach and Scheiner [36] in their respective studies.

With regard to our biophoton emission study, it can be stated that this experimental setup is entirely novel and distinctive. It is designed to map the alterations in plant life processes induced by the insecticidal active ingredients under analysis from an alternative perspective. Regarding our biophoton emission study, it can be stated that this experimental setup is entirely novel and distinctive insofar as it seeks to map the alterations in plant life processes resulting from the insecticide active ingredients under examination from an alternative perspective. There is a paucity of international studies that have addressed the changes that insecticides have on plant life processes. It can be reported that the delayed fluorescence values of plant individuals exposed to insect damage and treated with the tested active ingredients have increased. These increased DF values have been corroborated by Keszthelyi et al. [31] and Gerbovits et al. [16] in their respective studies. The researchers confirmed that systemic insecticide active ingredients that interfere with plant life processes lead to a more favourable plant health status. Some studies [22,23,24,25] have indicated that higher delayed fluorescence values of a healthier plant condition. The results of our study and observations lend support to this hypothesis. The lowest DF values were recorded for plants that had not been treated with insecticide and that had been subjected to intensive feeding by the red imported fire ants for 120 h.

The results of the UWLE analysis indicated that flupyradifurone and cyantraniliprole, when applied acropetally and basipetally, respectively, to oilseed rape plants that had not sustained insect damage, resulted in incremental growth. Some studies [23,37,38] have corroborated the assertion that UWLE measurements are augmented by the advent of stress conditions in plants. These findings indicate that insecticide active ingredients, when applied in different ways, may interfere with plant metabolism and act as stress factors. Conversely, the cyantraniliprole tested acropetally without larvae oilseed rape exhibited diminished UWLE values, suggestive of a more robust plant condition. The stimulating effect of the active substance cyantraniliprole on plant metabolism when applied acropetally has already been highlighted by Keszthelyi et al. [31]. Rastogi and Pospíšil [20] and Prasad et al. [38] found that an increase in ultraweak bioluminescence indicates oxidative stress in *Arabidopsis thaliana* L. plants, with lower values indicating a healthier plant condition. It was found that an increase in ultraweak bioluminescence indicates oxidative stress, with lower values indicating a healthier plant condition.

## 5. Conclusions

The experimental results provide substantial support for the activity of third-generation insecticidal agents, flupyradifurone and cyantraniliprole, in terms of both acropetal and basipetal translocation, in the context of controlling *A. rosae*. The utilisation of these compounds as seed treatments may present a viable strategy for the effective control of phytophagous pests. This will contribute to a reduction in the necessity for the repetition of crop spraying, thus effectively reducing the quantity of pesticide active ingredients used and applied per unit area. The deployment of this technology will facilitate the implementation of an efficacious IPM (Integrated Pest Management)-compliant control method. Nevertheless, the realisation of these future objectives will necessitate further impact studies.

## Figures and Tables

**Table 1:** The statistical relationships between different insecticide treatments and measured mortality values (*p* < 0.05). Significant differences between the values marked with *.

two-way ANOVA	**Treatments**	**DF**	**F**	***p***	**Mean** **(Mortality %)**
	active ingredients/time	1	50.061	<0.0001 *	flupyradifurone basipetal: 59.77% acropetal: 53.33%
	basipetal/time	1	35.142	<0.0001 *	
	acropetal/time	1	29.005	<0.0001 *	cyantraniliprole basipetal: 64.17% acropetal: 54.166%
	cyantraniliprole/flupyradifurone	1	0.576	>0.05	

**Table 2:** Comparison of different insecticide treatments of delayed fluorescence values resulting in two-way ANOVA (*p* < 0.05). Significant differences between the values marked with *.

		**DF**	**F**	***p***	**Mean (cps/mm^2^)**
without larvae	flupyradifurone acropetal/flupyradifurone basipetal	1	5.405	<0.04 *	567.42:350.83
	cyantraniliprole acropetal/flupyradifurone acropetal	1	2.621	>0.05	392.70:567.42
	cyantraniliprole basipetal/flupyradifurone basipetal	1	1.876	>0.05	422.46:350.83
with larvae	Untreated/flupyradifurone acropetal	1	5.388	<0.04 *	300.53:549.39
	Untreated/flupyradifurone basipetal	1	6.721	<0.02 *	300.53:539.96
	Untreated/cyantraniliprole acropetal	1	0.972	>0.05	300.53:461.74
	Untreated/cyantraniliprole basipetal	1	6.897	<0.03 *	300.53:382.12
	flupyradifurone acropetal/flupyradifurone basipetal	1	0.005	>0.05	549.39:539.96
	cyantraniliprole acropetal/cyantraniliprole basipetal	1	0.936	>0.05	461.74:382.12

**Table 3:** The statistical relationships of different insecticide treatments of ultraweak bioluminescence values (*p* < 0.05). Significant differences between the values marked with *.

two-way ANOVA	**Treatments**	**DF**	**F**	***p***	**Mean (cps/mm^2^)**
	Untreated/active ingredients	3	313.53	<0.0001 *	0.28:0.40
	flupyradifurone acropetal/cyantraniliprole acropetal	1	270.959	<0.0001 *	0.39:0.56
	flupyradifurone basipetal/cyantraniliprole basipetal	1	757.901	<0.0001 *	0.46:0.25
	flupyradifurone acropetal/cyantraniliprole basipetal	1	314.476	<0.0001 *	0.39:0.25
	flupyradifurone basipetal/cyantraniliprole acropetal	1	94.435	<0.0001 *	0.46:0.56
	cyantraniliprole acropetal/cyantraniliprole basipetal	1	531.282	<0.0001 *	0.56:0.25
	flupyradifurone acropetal/flupyradifurone basipetal	1	255.704	<0.0001 *	0.39:0.46

## Data Availability

The datasets analysed during the current study are not publicly available due to private proprietary but are available from the corresponding author upon reasonable request.
